# Minimal residual disease in prostate cancer patients after primary treatment: theoretical considerations, evidence and possible use in clinical management

**DOI:** 10.1186/s40659-018-0180-9

**Published:** 2018-09-04

**Authors:** Nigel P. Murray

**Affiliations:** Circulating Tumor Cell Unit, Faculty of Medicine, University Finis Terrae, Av Pedro de Valdivia 1509, Providencia, Santiago, Chile

**Keywords:** Prostate cancer, Circulating tumor cells, Tumor cell dissemination, Dormancy, Micrometastasis, Phenotype, Treatment

## Abstract

Minimal residual disease is that not detected by conventional imaging studies and clinically the patient remains disease free. However, with time these dormant cells will awaken and disease progression occurs, resulting in clinically and radiological detectable metastatic disease. This review addresses the concept of tumor cell dissemination from the primary tumor, the micrometastatic niche and tumor cell survival and finally the clinical utility of detecting and characterizing these tumor cells in order to guide management decisions in treating patients with prostate cancer.

## Background

With the worlds demographic changes and aging population prostate cancer has become the most common non-skin cancer in developed countries, 1,094,916 new cases were diagnosed and 307,481 deaths were reported worldwide in 2012 [1]. The natural history of untreated prostate cancer is one of evolution to a metastatic disease, especially disseminating to bone, over a variable time period. With advent of prostate cancer screening using the prostate specific antigen (PSA) there has been a migration to earlier stage cancers localized to the prostate gland [2]. Radical prostatectomy (RP) is a standard treatment option for these patients; however, 4–32% of these men with eventually relapse following radical prostatectomy (RP) [3–5]. In patients who achieve a PSA nadir of < 0.01 ng/ml post-surgery the failure of curative surgery is hard to explain. Although the peak time to relapse is 2 years, the majority will do so within 5 years [6, 7] but many patients remain clinically disease free for years until there is an increase in the serum PSA or overt metastasis are detected. One in five men have disease recurrence after 5 years and one in twenty after 10 years [6, 7].

Although an erroneous pathological classification of the tumor; in terms of either the cancer penetrating the prostate capsule (pT3) or an anatomically incorrect dissection plane (unrevealed positive margin), which left behind microscopic amounts of PC which subsequently progressed may explain some cases, this is not the case in the majority. The presence of sub-clinical micrometastasis (mM) not detected by conventional imaging is a more logical explanation of these cases. A positive bone scan has been reported in between 6 and 9% of patients with biochemical failure; however most of these studies are more than 15 years old, with median PSA levels of over 5 ng/ml [8, 9]. Similarly CT scanning fared little better with a detection frequency of 14% [8]. Since 2013 the use of Gallium-68-prostate specific membrane antigen (^68^Ga-PMSA) position emission tomography/computed tomography (PET/CT) has changed clinical practice and is incorporated in the Australian guidelines for prostate cancer restaging after biochemical failure [10]. It has a specificity of over 98% for prostate tissue; however the sensitivity is dependent on PSA levels. With PSA levels between 0.05 and 0.09 ng/ml 8% of patients had a positive PET/CT; 23% in the range 0.10–0.19 ng/ml and rising to 58% of patients with a PSA level of 0.20–0.29 ng/ml [11]. The 50% positive detection rate in patients with a PSA of 0.2–0.5 ng/ml is similar across differing studies [12, 13]. However, a systemic review of 37 published studies found a positive scan rate of 11–75% in patients with a PSA level of < 0.5 ng/ml [14]. Importantly this resulted in significant changes in the management of patients, in terms of local versus systemic rescue therapy in 29–87% of patients [14]. Limitations of the test include the 10% of prostate cancers that do not express PMSA [15] and nonspecific labeling of lymph nodes, especially those with follicular hyperplasia [16, 17]. However, with these advances there are more patients with “less indemonstrable minimal residual disease”.

Although new techniques are detecting smaller micrometastasis, there is a limit to image resolution, the undetected microscopic foci not removed by curative surgery are termed minimal residual disease (MRD) previously called micrometastatic disease. Minimal residual disease was first used to describe patients with hematological malignancies in complete clinical and hematological remission post bone marrow transplant yet using molecular techniques such as polymerase chain reaction had small numbers of leukemic cells detected in bone marrow. The term has been used increasingly in patients with solid tumors, especially breast cancer [18–20]. Minimal residual disease encompasses residual tumor cells which can persist locally as cancer stem cells, in the circulation as circulating tumor cells and in distant organs such as bone marrow as disseminated tumor cells or micrometastasis, the three faces of minimal residual disease [21].

The following databases were systemically searched during January 2018; Pubmed, Medline, SCOPUS, Web of Science, no language restriction, date restriction or publication status restriction were used. The reference lists of all included articles were hand checked for additional relevant articles not identified in the database searches. Full text articles were retrieved for any articles deemed potentially eligible.

## Primary dissemination

The metastatic process by which cancer cells disseminate from the primary tumor, survive in the circulation, implant in distant tissues, survive and grow is multistage and complex. To explain the presence of treatment failure in men with pathologically organ confined prostate cancer dissemination of tumor cells must be an early event, prior to treatment.

Circulating tumor cells (CTCs) were first described in 1869 by Ashworth [22] although only in the last few decades methods have been developed to detect these cells, defined as primary (pre-treatment) or secondary (post curative therapy) circulating prostate cells (CPCs).

Tumor cells are thought to enter the circulation passively, actively or both [23], as single cells, in clusters, in strands or in single files of cells.

Passive entry into the circulation occurs as a result of primary tumor growth, mechanical forces or friction which causes the cells to enter the circulation. Little is known about passive entry into the circulation, it has been postulated that cancers induce new blood vessel formation by the secretion of vascular endothelial growth factor (VEGF), this process of angiogenesis often results in leaky vessels as a consequence of weak interconnections of the endothelial cells and intercellular openings [24]. The endothelial cells do not form a normal monolayer and as such do not have a normal barrier function [25]. Thus with tumor growth, single or clusters of cancer cells may be pushed through these leaky intercellular openings and enter the circulation. This leakiness may be enhanced by the secretion of inflammatory mediators and the migration of leukocytes through the vessel wall [26]. The secretion of inflammatory cytokines which increase these endothelial openings [27] is one explication why epithelial cells may be detected in non-malignant disease [28, 29].

Passive dissemination may also occur as a result of tumor manipulation, either during surgery [30, 31], seed implantation during brachytherapy [32] and prostate biopsy [33]. Tumor cells may be passively moved through micro-tracks created by other tumor cells that are actively migrating into the circulation, as a result of proteolysis [34].

Active dissemination of tumor cells requires specific phenotypic characteristics which confer the ability to the tumor cell to detach from the surrounding cells, survive free of them, migrate towards the blood vessels where they cross the capillary endothelial wall to enter the circulation. Epithelial cells are anchored to other cells via adhesion molecules such as cadherins, claudins and plakoglobin. Normal epithelial cells show plasticity and undergo dynamic and reversible transitions between epithelial and mesenchymal cell phenotypes [35]. This epithelial to mesenchymal transition (EMT) is seen during embryogenesis, wound healing and tissue regeneration [36]. Cancer cells exhibit a decreased expression of anchor proteins such as E-cadherin [37–39] and beta-catenin [37], a loss of cytokeratins and EpCAM (epithelial cell adhesion molecule) [40–42] with upregulation of mesenchymal markers such as vimentin, N- and O-cadherins [43, 44]. E-cadherin is a calcium dependent cell–cell adhesion molecule, essential in maintaining the cellular polarity and architecture; its dysregulation modulates various signaling mechanisms including Wnt [45], RhoGTPase [46] and NF-*k*B pathway [47].

Single cells have been shown to exhibit these EMT changes while cell clusters detected in the blood only show a partial EMT, permitting them to enter the circulation while retaining some of the cell-to-cell interaction profiles of epithelial cells [48]. EMT can be initiated by paracrine signaling of TGF-Beta, WnT, platelet derived growth factors and interleukin 6 [35, 49] which in turn trigger the activation of the transcription factors Snail, Twist and Zeb thus maintaining the phenotype of a mesenchymal cell in an autocrine fashion [35].

There are also changes in matrix metalloproteinase (MMP) expression, especially MMP-2. These zinc containing endopeptidases are activated in situ and degrade the extra-cellular matrix, facilitating cell migration and invasion. Increased MMP-2 expression has been reported in primary prostate cancer and associated with an increasing Gleason score and pathological stage [33, 50, 51].

Not all cancer cells that actively migrate to the blood show EMT characteristics, centrosome amplification has been reported to induce cancer invasion [52]. In these cells cellular adhesion is reported to be decreased downstream of Rac-1 by an increased Arp2/3 dependent actin polymerization [52].

## Survival in the circulation and implantation in distant tissues

In order to implant at distant sites, CPCs must survive in the circulation, it has been suggested that only 0. 01% of CTCs can produce a single bony metastasis [53, 54], and injected CPCs obtained from men with castrate resistant prostate cancer may fail to produce metastasis when injected in immune compromised mice [55]. Sheer stresses found in the blood decrease the number of CTCs, however it has been reported that cells that have undergone EMT are more resistant than epithelial cells [56]. They resist anchorage dependent cell death, anoikis, which may be due to over-expression of anti-apototic proteins such as Bcl-2 [57] or suppression of caspase associated death via the activation of tropomyosin related kinase B [58].

Escape from the immune system may be direct, increased CD47 expression, an anti-phagocytic signal expressed on cancer cells prevents macrophage and dendritic cell attachment and the expression of pro-phagocytic calreticulin is decreased [59]. Furthermore, myeloid derived suppressor cells facilitate cancer cell survival by adhering to the CPCs [60]. In addition, CPCs become coated by platelets, transferring MHC class I antigens to the tumor cell surface. This coating of phenotypic normality disrupts the normal recognition of tumor cells by NK and T cell mediated immunity and as such improves tumor cell survival [61]. This platelet coating also enhances binding of the tumor cell to the endothelial lining of vessels at distant sites, enhancing invasion [62].

## The pre-micrometastatic niche and CTC homing

In 1889 Paget reported that the process of metastasis did not appear to occur by chance and suggested the “seed and soil” hypothesis [63]. Thus the seed (CTC) arising from a specific tumor shows a strong preference for the soil of specific metastatic sites, in the case of prostate cancer cells bone [64, 65]. Tumor cells may express parathyroid hormone related peptide (PTHrP) [66], chemokine CXCL 12 receptors, such as CXC chemokine receptor type 7 [67] or type 4 [68]. CXCL 12 is produced predominately by a diversity of bone marrow stromal cells, the cancer cells homing into the bone marrow by a CXL 12 gradient. In the bone marrow microenvironment there is a dynamic balance between stem cells, progenitor cells, mature immune cells and supporting stromal cells, this has been termed the metastatic niche [69, 70]. It is thought that there are two primary niches; the osteoblastic niche comprised of hematopoietic stem cells and the perivascular niche comprised of mesenchymal stem cells [70, 71]. In addition trophic factors, cytokines and chemokines act as bone marrow stromal mediators in the bone marrow niche. CXCL 12, integrins, osteopontin, vascular cell adhesion molecule-1 (VCAM-1), transforming growth factor beta (TGF-beta) and the receptor activator of nuclear factor kappa-b ligand (RANKL) are have been reported to influence the metastatic niche specificity for tumor type [69, 72]. Cell to cell adhesion is crucial for the initial seeding to the bone marrow niche. The expression on the surface of CTCs of integrin αvβ3 promotes the adherence to the extracellular matrix, via osteopontin, fibronectin, vitronectin and thrombospondin [73]. CTCs have also been shown to express α4β1 integrin which binds to the intercellular adhesion molecule-1 (ICAM-1) and VCAM-1 expressed by bone marrow and vascular cells [73]. Annexin II a protein that mediates the adhesion of hematopoietic stems to osteoblasts has also been reported in prostate cancer seeding to bone marrow [74]. Recent studies report that CTCs locate to the perivascular niche where endothelial cells, CXCL 12 abundant reticular (CAR) cells and mesenchymal stem cells regulate the implanting tumor cells [75]. Inversely there is a subpopulation of mesenchymal stem cells which carry endothelial and pericyte markers which suppress the homing of CTCs to bone marrow [76]. CPCs home in the niche via a SDF-1 cytokine gradient, SDF-1 is expressed in vascular “hot-spots” corresponding to regions in the bone that attract circulating tumor cells. The SDF-1/CXCR-4 interaction is pivotal for the recognition and binding to permissive vasculature [77]. The role of tumor suppression genes/proteins is also involved, CD82 expression on CPCs, the product of the tumor suppressor gene KAI1, impedes adhesion of the tumor cell to endothelial cells by inhibiting crosstalk with the Duffy antigen receptor [78]. The presence of CPCs that express CD82 is associated with low grade prostate cancer and the absence of bone marrow micrometastasis [79].

## Cancer cell implantation and survival

Although mechanical entrapment may be one mechanism by which CTCs lodge in distant sites it is insufficient [80], tumor cells must adhere to the vascular endothelium and extravasation by an active process. The initial attachment is via selectins, the presentation of selectin ligands is thought to be crucial to extravasation, especially E-selectin [81]. This results in morphological changes in the tumor cells, reorganization of the cytoskeleton and tyrosine phosphorylation [82]. This suggests that downstream signaling effects occur as a result of cellular adhesion. The expression of selectin ligands varies with tissue type and thus may influence the site of cancer cell colonization and explain in part organotropism [83]. This initial adhesion via selectins is reinforced by other adhesion molecules, the expression of immunoglobulin cell adhesion molecules ICAM and VCAM have been implicated in this role [84].

Once implanted, the biochemical signature of the niche will determine the fate of the cancer cell and it is thought to be the rate limiting step of metastasis [85]. In order to implant it is postulated that the tumor cells undergo a process opposite to the initial EMT, that of the mesenchyme epithelial transition (MET). It is suggested that there is re-expression of epithelial markers and down regulation of mesenchyme markers, which permits tumor cell adhesion and colonization in the new environment. The evidence for MET is more limited that EMT; it has been shown that E-cadherin expression is increased with respect to the primary tumor [86] and its re-expression may allow the cancer cell to survive in the target tissue [87]. The expression of E-cadherin in metastatic tissues may be found in patients with E-cadherin negative primary tumors [86]. Down regulation of E-cadherin in invasive cancer is due to promoter methylation and transcriptional repression and regulated by epigenetic mechanisms [88]. Re-expression of E-cadherin is not a random process; studies using breast cancer cell metastatic models in liver suggest that E-cadherin is directly regulated by the hepatocytes [89]. The methylation of a CpG island proximal to the E-cadherin transcription start site is inversely related to E-cadherin expression [90]. This is not the result of global hypo-methylation but specifically at the E-cadherin promoter site [89]. Re-expression secondary to hypo-methylation in prostate cancer cell models has also been shown to be driven by lung parenchymal cells [91]. Thus regulation of E-cadherin expression is not a result of gene loss or mutation, this epigenetic regulation allows for an increased phenotypic plasticity and influenced by factors in the microenvironment. Inhibition of Epithelial Growth Factor receptor signaling causes re-expression of E-cadherin in cultured prostate cancer cells [92] via the transcription factors Snail [93] and/or Slug [94] is one described mechanism, the second being by direct interaction at the promoter site or via the transcriptional factors Snail, Slug and Twist [95]. While Laminin-1, a component of the extra-cellular matrix, induces E-cadherin expression in 3 dimensional cultured breast cancer cells by inhibiting DNA methyltransferase 1 and reversing promoter methylation status [96].

However the MET is only partial, re-expression of E-Cadherin does not completely suppress the expression of the mesenchymal markers Vimentin and FSP1 [97], thus retain abilities for trans-endothelial migration [84].

Tumor-stromal cell interactions are important; in occupying the hematopoietic stem cell (HSC) niche tumor cells interact with bone marrow osteoblasts. The binding of tumor cells to bone marrow osteoblasts induces TANK binding kinase 1 (TBK1) expression that leads to inhibition of mTOR signaling and cell cycle arrest. Various cytokines and chemokines produced by osteoblasts determine the proliferative activity of the implanted tumor cell.

Growth arrest specific gene 6 (GAS6) is a growth factor that regulates cell cycling of HSCs and is expressed by osteoblasts, it acts as a ligand for the AXL, TYRO3 and MERTK family of tyrosine kinase receptors [98] inhibiting tumor cell proliferation through G_1_ cell cycle arrest and S cell cycle phase delay [99]. GAS6 overexpression activates MERTK via phosphorylation leading to a decreased p-ERK/p-p38 and increased cell cycle inhibitors/dormancy associated transcription factors p27, NR2F1, SOX2 and NANOG [100]. In the presence of GAS6 there is an increased AXL/TYRO3 receptor ratio that increases growth arrest, changes in this ratio of receptor expression changes the cells ability to enter or exit dormant or proliferative states [101]. GAS6 binds to the TAM receptor Axl on prostate cancer tumor cells which in turn induces expression of TGF-β1 and β2, this stimulates paracrine secretion (from osteoblasts) and autocrine secretion (from tumor cells) and leads to tumor cell dormancy through up-regulation of p27 an ubiquitous cell cycle inhibitor [102].

Thus GAS 6 appears to be important in tumor cells remaining dormant in the bone marrow niche and thus viable for extended periods. There is also evidence that GAS6 increases the number of prostate cancer cells with a stem cell phenotype, which is CD133 positive/CD44 positive, within the bone marrow [103]. Cancer stem cells (CSCs) are proposed to be stem like cells found in tumors and possess the capability to self-renew and differentiate into new diverse tumor cells. They represent a subpopulation of tumor cells that express specific surface antigens and possess mesenchymal phenotypes. The hematopoietic niche has the molecular mechanisms to regulate stem cell quiescence and self-renewal. Using murine models of human metastasis, it has been shown that of prostate cancer tumor cells recovered from bone marrow was significantly enriched for CSCs [104]. The expression of CD133 and CD44 was used to identify CSCs, increases in cytokine levels in bone marrow after intra-cardiac injection of tumor cells quickly returns to basal levels, using BrdU labeling CSCs had a lower proliferation rate compared with non-stem cell tumor cells, nor was there evidence that there was selective homing of CSCs or increased survival in the circulation [104]. It was further shown that direct cell-to-cell contact of prostate cancer cells and osteoblasts causes a significant shift from non-CSCs to CSCs [104]. GAS6 regulates part of the conversion of tumor cells into stem cells via its receptor Mer that activates the mTOR signaling pathway following cell to cell contact [104]. Furthermore, GAS6 inhibits the cleavage of caspase-3 and PARP to prevent apoptosis of the tumor cell [99]. When tumor cells are cultured with GAS6-null osteoblasts the conversion to CSCs is significantly diminished, and in mice models CSCs are found in much higher numbers in endothelial bone surfaces expressing GAS6 [104]. These changes to form CSCs are seen only in bone marrow and not in lung or spleen [104] and as such the bone marrow plays an important role in the accumulation of self-renewing, slowly proliferating CSCs. The growth of CSCs in the bone marrow depends on the GAS6 pathway, not only its expression in osteoblasts but also in prostate cells [101]. Consistently when prostate cancer cells reach the bone marrow Axl expression in prostate cells and GAS6 expression in osteoblasts both increase simultaneously [105]. The implication is that stromal cell-tumor cell contact converts the implanted tumor cells into cancer stem cells, which have the capability to self-renew and are resistant to chemo and radiotherapy.

It has also been reported that the microenvironment also decreases the expression of matrix metalloproteinase-2 (MMP-2). CPCs have been shown to express membrane MMP-2; tumor cells detected in bone marrow aspirates may also express MMP-2; however, on implanting in bone marrow the micrometastasis from low grade tumors and surrounding stromal cells are negative for MMP-2 expression, while in higher-grade cancers the micrometastasis retain MMP-2 expression [106, 107]. MMP-2 is important in the ability of cells to disseminate and in the activation of MMP-9 which leads to neovascularization [106]. The authors suggested that stromal Tissue Inhibitor of Metalloproteinase-2 might be responsible for this finding. Decreased MMP-2 expression together with increased epithelial cell marker expression by tumor cells decreases their ability to further disseminate.

In order to grow, the tumor cells need space within the micrometastatic niche. The Receptor Activator of Nuclear Factor Kappa B-Ligand (RANKL) is expressed by osteoblasts and stromal cells within the bone marrow. RANKL activates osteoclastogenesis that leads to bone reabsorption and creates space for the tumor cells. Osteoclastogenesis causes demineralization and the release of tumor growth stimulating factors from the extracellular matrix [108]. RANKL released from local osteoblasts stimulates the expression of interleukin 6 (IL-6) in the tumor cells. IL-6 activates three major signaling pathways, the Janus tyrosine family kinase (JAK) signal transducer and activator of transcription (STAT) pathway, the ERK1/2 and MAPK pathway and the PI3-K pathway. These signaling pathways regulate apoptosis and thus cell survival and cellular proliferation and play a key role in bone metastasis [109]. The secretion of IL-6 from tumor cells induces bone turnover and enhances osteoclastogenesis and osteoblast differentiation [110] which in turn leads to production of IL-6 by osteoblasts and further stimulates tumor cell proliferation in a paracrine fashion [111]. The IL-6 expressed by tumor cells stimulates the expression of RANKL and increases tumor cell sensitivity to its effects [112]. The inhibition of IL-6 production with tocilizumab decreases skeletal tumor growth, serum RANKL levels and RANK expression in animal models [112].

## Escaping from dormancy

Little is known on how cells escape from dormancy, many hypotheses have been proposed on how tumor cells are maintained in a dormant or indolent state before the emergence of overt metastasis. The lack of angiogenesis, immune surveillance by T-cells, balanced proliferation and apoptosis have all been proposed [113]. The majority of patients have tumor cells negative for Ki-67, a marker for cellular proliferation [114], although the fraction of Ki-67 positive cells in higher in more aggressive cancers [115]. More recently there is experimental evidence using mouse models that aberrant unregulated expression of the vascular cell adhesion molecule-1 (VCAM-1) is involved in the progression from indolent to overt metastasis [116]. It is thought to recruit pre-osteoclasts to the bone marrow micrometastasis and promotes signal flow through the P13K-Akt pathway and possibly dependent on an intact NFκB pathway [117]. More recently the identification of microRNAs (miRs) as regulators of the transcriptome are involved in this process. Sixteen miRs were found to be highly expressed in dormant tumors, down-regulation of these dormancy associated miRs was correlated to the switch to a fast growing angiogenic phenotype [117]. miR-580 and miR-190 expression was shown to be inversely reverted to disease stage. It is thought that loss of dormancy associated miRs switches tumor cells to a stage of exponential growth [117]. Two important targets of miR-580 and miR-190 are the EphA5 and Angiomotin genes, both are expressed in dormant tumors, are inversely related to tumor stage and down regulated during the angiogenic switch [118]. The circulating protein products of these two genes, EphA5 and angiostatin, respectively, are correlated with the tumor dormancy phase [118].

Once free from dormancy, there is tumor growth and the appearance of clinical and radiological evidence of metastasis.

## Changing the soil selects the seed—Paget revisited

Micrometastatic growth is seen in clinical practice as an increase in serum PSA after curative therapy and before any imaging studies show evidence of metastatic disease and is defined as biochemical failure.

First line treatment is with androgen deprivation therapy (ADT) which can be achieved by bilateral orchiectomy (surgical castration) or more frequently a luteinizing hormone-releasing hormone (LHRH) agonist or antagonist (medical castration) which appear to be equally effective [119]. Treatment success is reflected in a decreasing serum PSA, but after a variable time period the serum PSA increases although the serum testosterone remains at castrate levels (< 50 ng/dl) and defined as castrate resistant prostate cancer.

A fundamental question is whether the ADT resistant tumor cells are a result of clonal selection or clonal evolution as a result of genetic instability or both. In the case of clonal selection, the phenotypic and genotypic characteristics should be present in at least a subgroup of tumor cells in the primary tumor: The use of ADT gives this cells a selective advantage permitting them to proliferate and form metastasis. With clonal evolution the tumor cells may not be present in the primary tumor, but with time the genotype has evolved to an ADT resistant phenotype. There are few clinical reports of sequential changes with time, the majority are in animal models, or comparing ADT sensitive and resistant tumors after ADT.

In animal models ADT causes EMT with increases in the expression of N-cadherin, Zeb1, Twist 1 and Slug and decreases in E-cadherin. Although the tumors diminished in size, the surviving tumor cells had increased “stemness” and activated TGF-beta both at mRNA and protein expression levels, as well as N-cadherin and vimentin and decreased E-cadherin [120]. These changes have been observed in human prostate cancer tissue [121]. Zeb 1 appears to mediate androgen deprivation induced EMT via a bidirectional negative feedback loop with ADT and its inhibitor miR-200b decreases [120]. Over expression of Zeb 1 is sufficient to switch cells from a non-cancer stem cell to a cancer stem cell status and required for maintaining tumor cells in a stem cell state [122]. These cancer stem cells express the CD133 membrane protein as well as CD44. As to the question of the origin of these cells, it has been reported that the basal cells of prostate contain a subpopulation of androgen independent epithelial stems cells [123], furthermore it has been reported that prostate cancers contain both androgen dependent and independent tumor cells. The selective pressure of ADT causes clonal expansion of the androgen insensitive cells altering their relative frequency and leads to the development of castrate resistance [124]. Hormone free cell cultures obtained from early stage prostate cancer specimens showed that colonies of androgen independent cells grow in 70% of cases, supporting the hypothesis that clonal selection may be a key mechanism in castrate resistant prostate cancer [125].

In the clinical, the expression of HER-2 has been associated with resistance to ADT. Prostate cancer and bone marrow micrometastasis contained both HER-2 positive and negative cells, that the risk of treatment failure was similar in patients with HER-2 positive and negative micrometastasis. However, after starting ADT there was selection of HER-2 expressing cells, HER-2 negative cells being eradicated and these men had a higher risk of progressing to castrate resistant prostate cancer and a shorter time to treatment failure with ADT [126, 127].

## Mechanisms of androgen resistance

Patients treated with androgen/androgen receptor (AR) directed therapies, including abiraterone and enzalutamide have tumor cells with a molecular signature consistent with continued “addiction” to AR. These cells acquire or possessed molecular alterations in the AR axis. The AR gene is frequently amplified or mutated (62%) and less frequently there is amplification of the androgen receptor (< 1%). In primary prostate cancer specimens there are numerous reports of recurrent somatic mutations, copy number alterations and oncogenic structural DNA arrangements [128–130]. These include point mutations in SPOP, FOXA1, TP53, copy number alterations involving Myc, PTEN, CHD1 and transformation specific (ETS) fusions of which some have prognostic significance [131]. The combination of Myc activation and PTEN loss are sufficient to create genomic instability and lethal metastatic prostate cancer [132]. In men with castrate resistant prostate cancer genomic studies showed a high frequency of AR pathway alterations; this suggests that the tumor cells remain dependent of AR signaling for viability. In metastatic castrate resistant prostate cancer there is frequently over-expression of both full length AR (AR-FL) and AR variants (AR-V). AR-Vs are alternatively spliced isoforms of the AR mRNA, and lack the ligand binding domain, which is the intended target of all existing androgen/AR directed therapies. AR-Vs can activate AR signaling in the absence of androgens or the AR-FL. The levels of expression of AR-Vs are increased in castrate resistant prostate cancer, in response to AR blockade and associated with disease progression [133]. AR-V7 is the more frequently found variant and often co-expressed with AR-FL, the levels of nuclear AR-Vs required to drive an androgen-independent transciptome remains unclear. The levels of AR-V mRNA and protein expression relative to AR-FL varies within normal and malignant prostate tissues [133], CPCs [134] and prostate cancer cell lines [133]. The mechanism to achieve AR-V is unknown, rearrangements of the AR gene and/or changes in splicing dynamics have been suggested. AR-Vs not only activate transcription of AR regulated target genes such as PSA, HK2, TMPRSS2 and NK-X3-I [135] but also genes associated with the regulation of the cell cycle [136]. Over-expression of AR-V7 has been shown to be associated with higher levels of SNAIL, TWIST, N-cadherin and ZEB1 without affecting E-cadherin expression, with the suggestion that over-expression of AR-V s produces a partial EMT [137]. Similarly, the expression of AR-V3 was higher in Gleason 7–9 primary tumors, was shown to involved in inducing stem cell markers such as Nanog and Lin28B and EMT markers, and finally the use of enzalutamide led to increased AR-V3 expression [137].

As such it would seem that in the primary tumor cancer stem cells are present and disseminate, whether there is clonal selection or clonal evolution or both, and the relative importance of either remains unknown.

## Circulating tumor cell and micrometastasis detection

The first reports of bone marrow micrometastasis in men with prostate cancer used bone marrow aspiration samples, differential gel centrifugation to enrich tumor cells and immunocytochemistry with anti-cytokeratin antibodies to detect tumor cells [138]. The frequency of tumor cell detection depends on the method used, immunocytochemistry or RT-PCR and the marker, PSA, PMSA or cytokeratins. Detection of PSA mRNA using RT-PCR was not associated with the results of immunocytochemistry [139], is limited by the illegitimate transcription of tumor associated or epithelial specific genes in hematopoietic cells and the deficient expression of the marker gene in tumor cells [140]. Both immunocytochemistry and RT-PCR have similar specificities (PSA mRNA versus anti-PSA) but RT-PCR has a tenfold increased sensitivity at detecting micrometastasis [141].

It has been suggested that cells detected in bone marrow aspirates may not represent true “micrometastasis” but rather are prostate cells circulating in the bone marrow compartment, explaining the expression of similar phenotypic markers, as CPCs. “True” micrometastasis were those detected in biopsy specimens. The low concordance between prostate cells detected in bone marrow aspirates with those detected in biopsies for patients with Gleason 5, 6 and 7 suggests there is a difference in their physiological/oncological role. In high grade Gleason 8 and 9 there is good concordance between the results of the two methods of sampling. There are no studies of in vivo tumor cell rheology in the bone marrow. However, there are in vivo optical imaging studies in laboratory animals demonstrating the mechanisms of tumor cell attachment to the endostium that are similar to stem cell engraftment [142, 143]. Topological and chronological patterns of stem cell seeding have shown that most cells drift within the bone marrow space and then are gradually found close to the endosteal surface. The center of the bone marrow space seems to be the site of proliferation of the transplanted cells and not at the endosteal surface [144]. Further data has shown that the adherent cells are viable, whereas cells in transit contain a percentage of dead or dying cells [145]. Thus cells anchored to the endothelial surface may not be detected in bone marrow aspirates and thus explain partially why aspirate negative patients may relapse in the bone, or inversely why bone marrow aspirate negative patients go on to development bony metastasis. In high grade cancer the interchange between attached and in transit may be sufficiently high so as the results of aspirate and biopsy are concordant [106].

There are a number of techniques that have been developed for the detection of circulating tumor cells, which has hindered the comparison of different studies and the consensus of defining these cells. Each method has differing advantages and disadvantages and has been extensively reviewed [118, 146]. In summary because of the rarity of these cells, enrichment methods are used to concentrate CPCs. Density gradient centrifugation separates a layer of mononuclear blood cells and CPCs from other blood cells, it is a simple fast process but tumor cells may be lost during the process, as they sediment to the granulocyte fraction or when present as clusters sediment to the bottom of the tube. Due to the size differences between CPCs and normal blood cells filtration has been used as a method to enrich CPCs from whole blood. The OncoQuick^®^ system uses a porous barrier above the density gradient while the Screencell^®^ cyto, ISET^®^ and Metacell^®^ are three commercially available filtration systems. CPCs are isolated on the filter and then subsequently stained. The filter based systems do not detect CPCs smaller than 8 µm, and the filter may become clogged during the process [118, 147]. Leukapheresis of large blood volumes has been reported to detect CTCs in up to 90% of non-metastatic breast cancer patients, the authors also reported that in healthy controls there was a high background of cytokeratin positive CD45 positive cells due to false positive staining of leukocytes [148]. The FDA approved CellSearch^®^ system uses immunomagnetic selection of CTCs with anti-EpCAM (positive selection) while there are methods using depletion of CD45 positive (leukocytes) (negative selection). In high risk prostate cancer patients CPCs were detected in 37% of patients using CellSearch^®^, 55% with Cellcollector^®^, and 59% with Epispot^®^ [149]. The use of specific antibodies such as EpCAM to enrich CPCs results in the loss of CPCs which have undergone EMT.

## Clinical evidence and possible uses

Studies reported that the presence of micrometastasis was associated with tumor stage and Gleason score [150–152], however other reports did not confirm this finding [139, 153, 154]. Furthermore samples taken after radical prostatectomy or radiotherapy had a lower frequency of micrometastatic detection [153, 155, 156] and that these cells were cytogenetically aberrant [157]. The inference of these findings is either local removal of the primary tumor decreases or eliminates micrometastatic disease, that is to say that the micrometastasis is dependent on a factor produced by the primary tumor in order to survive or the method of detection in some way is deficient or the interpretation of what the test is detecting. Using bone marrow aspirate and biopsy samples it was shown that there was no difference in the frequency of micrometastasis detected pre-treatment but there was a significant difference post-treatment, there was a significant reduction in “micrometastasis” detected in bone marrow aspirates [158]. Phenotypic classification of circulating prostate cells, and cells detected in bone marrow aspirates were similar but differed from the phenotypic characteristics of prostate cells detected in bone marrow biopsies with respect to CD82 and MMP-2 [106].

The detection of prostate cells in bone marrow aspirate samples as a prognostic marker has given conflicting results; this may be in part due to a short follow up time. Some studies have reported no association with biochemical failure [159, 160], whereas others have reported a higher rate of failure when detected in post-treatment samples [156, 161].

There is more evidence for the prognostic role of secondary circulating prostate cells that is those detected after curative therapy. In patients with non-metastatic disease the presence of secondary CPCs is associated with early relapse [162–165]. Their presence was associated with a shorter PSA doubling time and shorter time to treatment failure [166]. EpCAM based detection systems failed to show an association with prognosis [167, 168] in men with localized prostate cancer. In contrast using telomerase based technology [169] or RT-PCR [170] an association as an independent prognostic factor was reported.

More recently, it has been reported that men CPC positive have a higher risk of early treatment failure, whereas those with only bone marrow micrometastasis have an identical failure rate to men negative for CPCs and micrometastasis up to 5 years of follow-up, after this time there is increasing failure in this group. This suggests that there are two types of minimal residual disease, one associated with a more aggressive outcome, that is CPC positive, and one showing features of dormancy and later treatment failure [171].

## As a guide to treatment options

Standard recommendations include the following; in men with positive surgical margins radiotherapy is suggested as adjuvant therapy to eradicate local foci of tumor left behind at surgery. At the time of biochemical failure, salvage radiotherapy or androgen deprivation therapies are alternatives. The use of PSA kinetics, time to relapse, PSA doubling time and Gleason score have all been proposed to define local or systemic failure. However, in this group of patients 67% were found to have bone marrow micrometastasis. In comparison with the anterior parameters, there was no association with micrometastatic disease. The detection of bone marrow micrometastasis implies the presence of systemic relapse and such systemic treatment [172]. First line ADT treatment is with a LHRH agonist or antogonist, there is normally a decrease in the serum PSA for a period of 3–5 years. Thereafter resistance to ADT develops with an increasing PSA and testosterone levels < 50 ng/dl, second line hormonal therapy using newer agents such as aberiterone or enzulatamide are used, and finally if failure continues the use of taxanes. This may be accompanied by the appearance of bone metastasis in imaging studies.

It has been shown that ADT can eliminate bone marrow micrometastasis in approximately 80% of patients [173, 174]. Further studies reported that micrometastatic cells expressing HER-2 were resistant to ADT and were selected in an androgen-deprived environment [125]. Thus although serum PSA decreased with ADT, a population of resistant cells were selected which later produced PSA failure. In contrast treatment with diethylstilbestrol eliminated both positive and negative expressing HER-2 cells, possibly by stimulating beta estrogen receptor and blocking HER-2 stimulation of the androgen receptor (AR) downstream [125]. The AR antagonist bicalutamide is effective in treating prostate cancer, irrespective of HER-2 expression levels [175]. The expression of HER-2 was similar in CPCs and bone marrow micrometastasis [125]. Thus the expression of HER-2 could be used to select the better treatment option. Continued AR activity in resistant cancer has been linked to the expression of a number of truncated but constitutively active AR isoforms. One such variant is AR-v7; classifying patients as CPC negative, and CPC positive AR-v7 negative and positive it was possible to determine three prognostic subgroups, CPC negative having the best prognosis, CPC positive AR-v7 positive the worst [176]. The frequency of CPCs expressing mRNA for AR-v7 increases with successive endocrine therapies [177], overall survival was superior with the use of taxanes in these positive patients. The expression of AR-v7 in CPCs is associated with resistance to abiraterone and enzalutamide but not resistance to cabazitaxal [178]. Using single cell immunofluorescence analysis, CPCs were predominately AR-on (AR activity positive) pre ADT, first line ADT produced a switch from AR-on to AR-off (AR activity negative) CPCs, whereas variable expression was seen after second line ADT. The presence of AR-mixed or increasing AR-on expressing CPCs while being treated with abiraterone was associated with a decreased survival [179]. Thus the possibility of determining the best treatment options using CPC phenotypic expression seems possible, as well as detecting resistance to treatment before detectable disease progression.

What is important is that CPC detection is method dependent, and as such there is no consensus on the best approach for their detection. Those methods relying on specific markers will not detect CPCs lacking the determined marker and this may be stage dependent and on the presence of EMT and MET.

## Conclusions

with advancing technology and single cell gene analysis the use of liquid biopsies of CPCs may be useful in the classification of patients, assess the risk of treatment failure in specific patients and which treatments may be more appropriate. In combination with the analysis of micrometastatic cells found in bone marrow, it may be possible to tailor treatment to eliminate these residual cells or maintain this cell population in a dormant state on an individual patient basis.
